# Putting the Spirit into Culturally Responsive Public Health: Explaining Mass Fainting in Cambodia

**DOI:** 10.1007/s10943-018-0661-8

**Published:** 2018-06-28

**Authors:** Maurice Eisenbruch

**Affiliations:** Department of Psychiatry, School of Clinical Sciences at Monash Health, Monash University, Clayton, VIC Australia

**Keywords:** Mass fainting, Cambodia, Buddhism, Possession, Spirits, Inauspicious death

## Abstract

The study explores the cultural and religious meaning behind episodes of mass fainting sweeping through garment factories in Cambodia. An ethnographic study was conducted at 20 garment factories in Kandal, Preah Sihanouk, Kampong Cham, Kampong Speu, Takeo, and Kampong Chhnang provinces. Informants were 50 women who fainted or possessed and their families, factory and clinic staff, and monks. Informants described their views on the causes of the mass fainting. Based on the informants’ views, the seeds were sown when factories were built on former Khmer Rouge killing fields, when local guardian spirits were disrespected and when the factories were not inaugurated with the proper rituals. We found that an inauspicious death, a conflict leading to violation of a vow, or culturally inappropriate interventions by management explained what triggered the episodes. The results show that people believe that mass faintings occur in parallel with tensions between the workers and the foreign owners of the factories and tensions between the human and spiritual owners of the land. The study has implications for the development of culturally responsive public health interventions in mass group phenomena.

## Introduction

This article argues for a culturally responsive public health response to mass events. The case in point is mass fainting, a global phenomenon of growing concern. Scores of people, usually women in crowded or cohesive settings, such as garment factories and schools, who are in acute fear of disaster, experience a sense of suffocation and then appear to faint (Eisenbruch 2017). It has been reported around the world and seems akin to recent group phenomena in Asia, such as the salt hysteria in China after the Fukushim disaster in Japan (Associated Press 2011) or the mass hysteria evacuation of Melbourne Airport (Bartholomew 2005) and perhaps to occupational epidemics in western societies (Hall and Morrow 1988; Reid et al. 1991).

The African literature in post-war contexts also seems relevant, for example, the work of Honwana (Honwana 1998) and especially Igreja (Victor 2003) who described how the Gamba spirits in Mozambique expressed social tensions after the Frelimo–Renamo conflict. De Jong and Reis’ (2013) work in Guinea Bissau depicts dissociation as a way of processing post-war traumatic stress in, and use the term ‘collective trauma processing’ to analyse how the local cultural idiom is a pathway to mitigate the consequences of political violence, and shows striking similarities to what is happening in the garment factories.

This article takes up the challenge of this literature, to chart with greater precision the local cultural understandings of the mass event. The study focuses on mass fainting in Cambodia, where there is a rapidly changing social scene due to neoliberal forces and a developing garment sector, which serves as the primary employer of women. At the same time, there is a deeply entrenched Cambodian form of Theravada Buddhism and folk beliefs involving the supernatural world. In this article I will show how the guardian spirits, perceived as ancestors who protect and control the land and its inhabitants and communicate with the living through mediums (Guillou 2012), are especially relevant to mass fainting. Even after war and economic development, these spirits may be displaced but maintain an ongoing presence (Arensen 2012). The inescapable element in Cambodia is the legacy of the Khmer Rouge era, and the dead of the mass graves can be as powerful and aggressive as the guardian spirits in responding to trespass (Guillou 2012) and human settlement (Arensen 2017).

This Janus-faced view of contemporary Cambodian society is associated with two parallel issues: the place of the supernatural in the Khmer-speaking world and public health issues in the non-Khmer-speaking world. The potential discrepancies in the discourse on these issues drive us to investigate the workers’ explanations for mass fainting. In 2013, the *Cambodia Daily* claimed that the English-language press underreports the importance of spirit possession (Wallace et al. 2013), but we confirmed that the Khmer-language press widely and consistently highlighted the role of spirits. When the Anful Factory was reopened with a Buddhist dedication, lawmaker Mu Sochua was critical that they were ‘looking for ghosts in Cambodia instead of sending in experts to examine workplaces … [T]hey said Cambodian women were suffering from hysteria, now they’re looking for ghosts’ (Nil and MacIsaac 2011). The Minister of Cult and Religion, which promotes formal religion and discourages ‘superstition’, was sceptical that the workers ‘have nothing else so they turn to this belief’ and that ‘local beliefs can be used by the management to their own advantage who, instead of improving working conditions, can just hold an offering ceremony and forget about everything else and get away with it’ (Menghun and Hruby 2013).

In an earlier report on my ethnographic study of the mass fainting of garment factory workers in Cambodia, I reported on cultural understandings, including the understanding that ghosts ‘haunted’ factory sites in the wake of the Khmer Rouge atrocities or recent fatal accidents and that guardian spirits at sites retaliated against foreign owners when there were certain violations (Eisenbruch 2017). Prefigurative dreams, industrial accidents, or the possession of a co-worker heralded episodes. Workers witnessing a co-worker fainting would feel afraid and also faint. When taken to clinics, some showed signs of continued spirit influence. Mass fainting seems to be an outcry of protest by disempowered workers whose misery is made more acute by the nature of their factory work. As Arnold (2017) notes, the mass fainting is a form of ‘political society tactics… when they reach the limits of civil society channels’ (p. 29).

Although there has been little work on the cultural meaning of mass fainting, there is a good understanding of the role of spirit possession in factories. During the early 1980s, newly industrialising Malaysia faced outbreaks of spirit possession among female factory workers on shop floors (Ong 1988). As is the case in Quimsa, production schedules in Malay factories are often disrupted by spirit attacks (Crain 1991). The corporate view, using the cosmopolitan medical model, labelled workers as deviants and patients when possessed and regarded the events as an intrusion of archaic beliefs in a modern setting (Ong 1988). As is the case in Cambodia today, the Malaccan Factory managers perceive the ‘real’ causes of outbreaks of possession to be physical (undernourishment) and psychological (superstitious beliefs). However, the spirits play a special role in expressing grievances and tensions in the face of extreme stress (McLellan 1991).

In contrast, there is strong anthropological evidence that occupational illness epidemics, such as states of possession, are employed against superiors when other forms of protest are blocked (Hocking 1987). Crapanzano (1977) highlight the ‘facticity’ of possession as an idiom of communication and semiotics that resolves conflict. Almost in anticipation of the fainting scenes in Cambodia, Ong (1988) argued that spirit possession in multinational factories in Malaysia are part of the complex negotiation of reality by an emerging female industrial workforce. Ong’s approach offers a useful starting point for deciphering how ‘affliction, gender symbolism, social boundaries, morality, cultural experience and hegemony in the process of social change’ are connected to mass fainting in Cambodian garment factories.

I will examine the cultural constructions of mass fainting beginning with an explanation of the phenomenon as a consequence of past legacies that blighted the factory sites because they were built without spiritual permission or were built on former Khmer Rouge killing fields, which themselves were blighted by ghosts of the dead. Mass fainting reflects the drama of industrial and spiritual disputes and other tropes of violence in which workers, for example, are pressured into illicit affairs at the factory. Then, there are ethnic and religious variations in the interpretation of violations that can lead to mass fainting, which necessitates rethinking the role of industrial fumes, and also variations in the social context of industrial conflicts, such as the consequences of broken vows. A discussion will then follow on issues involving factories that were established over moral violations and effects on the local ecology, such as sacred trees, which raises the question of the role of guardian spirits, in particular, the ‘Landlord of Water, Landlord of Earth’. These observations lead to a discussion on the Buddhist prototype for illness, which arises when the rights of the spiritual owners of the land are disregarded, and a discussion on how to implement appropriate interventions, taking into account the complex ethnicity of the factory owners and managers. In concluding this study, there is a brief discussion on factories affected by inauspicious and premature deaths from the Khmer Rouge era and the shadow of suicide.

## Methods

The present author is a Khmer-speaking medical anthropologist and transcultural psychiatrist and is assisted by a male Cambodian based in Phnom Penh who has undertaken fieldwork for the author for almost 25 years. The study was approved by the National Ethics Committee for Health Research (NECHR) in Cambodia.

The method is set out in detail in the earlier article (Eisenbruch 2017) and merely summarised here. Fieldwork was undertaken from 2009 to 2016 in 60 factories and in workers’ lodgings, health clinics and temples. Of the 60, 48 factories were selected in which fainting of some sort had occurred. Typically, the team arrived at the factory shortly after an episode and the women were encountered during the aftermath. There were some incidents where the team was present at the factory when the fainting occurred and could observe the affected individuals en masse. All individuals that were approached agreed to participate and no one dropped out of the study. The researchers were not involved in the patient care of those affected.

Meetings involved workers from any factory in which fainting took place, and an estimated total of 200 workers were included (precise numbers can be difficult to ascertain when in the midst of a fainting melee or a healing ceremony in the factory). Demographically, informants were women between 18 and 60 years of age, mostly Khmer, but some had Cham or Chinese-Khmer backgrounds. Upon learning of an incident, the team would arrive swiftly and was sometimes able to be at the scene during a fainting episode and to follow-up on any subsequent cascades. Informants included women who fainted and a few who were said to have been possessed.

We explored the informants’ attributions of cause, including predispositions and triggers. Attention was paid to the ways in which people connected the fainting with supernatural forces such as local guardian spirits and the ghosts of people who suffered inauspicious deaths during the Khmer Rouge period or during contemporary events such as industrial accidents at or near the garment factories. Informants were encouraged to share their beliefs about any violations of the customary codes of conduct.

Monks and healers were also interviewed as expert informants. We conducted fieldwork with a total of 20 monks and Buddhist ritual officiants.

Encounters took place through a free-flowing exchange in Khmer. Women who fainted were asked about their subjective experiences; health workers were asked the same question, but with greater attention on the connections they witnessed between local cultural and medical explanations for the fainting and associated treatment. Monks and healers were asked to expand on the taxonomy of fainting and on the Buddhist, mystical, animistic, and magical frameworks that helped them understand and treat it. The ethnography included participation in ritual processes that ran for up to 3 days and through follow-up interviews, through meeting participants at calendrical ceremonies and through exploring their experiences after the initial episode through any further episodes.

All fieldwork was conducted in Khmer and translated and transcribed into English. Data were collected in the course of brief encounters during the episodes of mass fainting and in the therapeutic rituals performed in the aftermath. We encouraged the informants, once we built trust and they felt more comfortable, to share their views of the supernatural world they understood to be involved in the mass faintings rather than confining themselves to the popular medical views they thought we wanted to hear.

Audio and video recordings were analysed in Khmer, and three-quarters of the recordings were translated into English. Khmer terms are presented where the local idioms have no precise English equivalent and are spelt out using Huffman’s adaptation of the IPA phonetic transcription (Huffman et al. 1970) rather than transliteration, to help non-speakers of Khmer to more easily and consistently pronounce the terms.

## Results

The media say that the fainting is caused by vitamin, glucose, or calcium deficiencies; toxic odours; chemicals ingested or inhaled; insecticides used liberally in the factory; or crowded and sweltering factory floor conditions. The workers focused on their poor diet consisting of ‘Number Three’ food, which is the barely edible, cheapest food sold outside the factory gates. Clinic staff viewed the causes as ‘fever of the intestine’, ‘iron deficiency’ and ‘deficiency of red blood’. The starting point, provided by the doctor, once unravelled in the minds of the local villagers, shows how to place the biological determinants in a more accurate cultural field. Overall, most informants, even if they *knew* about the public health messages on the causes of mass fainting, *believed* it to reflect the fact that factories were built without regard for the local spirits. It was worse when the factories were constructed on the sites of the former killing fields, when factories were contaminated by recent inauspicious deaths and when workers had violated moral codes.

### Blighted Legacies

#### Land

The factories were built on land believed to be owned by guardian and tutelary spirits specific to each factory site, with each territory’s spirits having been established from time immemorial.

The Tiger Wing Factory and Eternity Global Sporting Factory were said to have been erected on Khmer Rouge killing fields. In violation of the guardian spirits, developers grabbed the land and demolished the house of the guardian spirit. Informants believed that a failure to seek permission from the local tutelary spirits (see Table 1) or from the generic ‘Landlord of the Water, Landlord of the Earth’, could lead to mass fainting.

**Table 1:** Tutelary spirits at factory sites

Factory	Tutelary spirits at the factory locale
Heart Enterprise	Honourable Mister Twin (*cah srok plʊəh*)
M and V	Guardian Spirit of the Water Lily Pond (*trɑpeaŋ skɔǝn*)
Inter Hopewell	Varnish Tree Guardian Spirit (*traaŋ*)
Yak Jin	Madam Yiey Grandma Dup
	Madam Very Happy
Tiger Wing	Male Guardian Spirit *Schleichera edulis* (*pʊəŋrɔɔ*)
	Old Madam Pond Salvadora capitulata (*trɑpeaŋ snaay*)
	Elves in the Dead End Forest (*mrɨɲ kʊəŋviel prey kɑmbot*)
Eternity Global	Pŋieh (tree used for coffin lid) Guardian Spirit (*neak taa pŋieh*)
Sabrina Manufacturing	Old Lady White and Old Lady Black (*look yeay sɑɑ look yeay kmav*)

Sometimes, the guardian spirits demanded offerings. If not given spirit food, they retaliated by causing a mass fainting episode. Typically, people had to organise a ceremony to pinpoint what the spirit was after so they could stop the problem. The workers had no bargaining power with the factory owner, except to issue threats to shut down the factory for a few days after a mass fainting.

Even foreign factory owners sometimes came to realise the importance of the spirits in mass fainting episodes. At the Tiger Wing Factory in 2010, when hundreds fainted, the Chinese owner understood the faintings to be a demand by the local spirit ‘Landlords’ for compensation for the destruction of their territory and encouraged people to build shrines. According to our follow-up, Tiger Wing Factory has since been free of mass fainting episodes.

#### Inauspicious Deaths

Villagers would sometimes stumble across bones from the days of the Khmer Rouge. The corpses of those who died inauspiciously became wandering spirits. Permission had not been sought for the executions that occurred on the land controlled by the ‘Landlord of the Water, Landlord of the Earth’, where the Tiger Wing Factory was later built. Worst of all, it was believed, the factory was built on the site of a Khmer Rouge mass grave. This was no place to put garment workers. The public health message was that faintings were caused by chemical glue in the factory, but some workers suspected that the stench of the glue was the stench of the ghosts.

Older workers had their traumas reanimated through episodes of mass fainting. Grandma Thida, aged 49, was a janitor at the Tiger Wing Factory. Her mother was Khmer and her father Chinese-Khmer. In 1975, the Khmer Rouge force-marched her family to Kampot province and killed most of them. Thida witnessed blood seeping from the corpses. Terrified that she would be next, she forced herself to be resolute and to concentrate on survival.

One morning, a woman at Tiger Wing became possessed and triggered mass fainting. The panic-stricken bystanders ran to Thida, who uttered strange sounds which, she later said, were the howling of Khmer and Chinese-Khmer wandering ghosts (*taay haoŋ*) of those killed by the Khmer Rouge. The onlookers told her that she had spoken in fragments of Chinese, Japanese and Thai. Through her voice, the ghosts complained, ‘Make us a ghost house! You took all of our land to make a factory!’ They bargained with the owners that once they got their way, they would release the workers from their fainting and let them get back to work.

Episodes were heralded by a bad omen or horrifying dream. One night, Thida saw ‘Old Lady of the Toothbrush Tree Pond’ appear in a dream and demand fruits, and the next morning while at work, Thida fell into a trance. She saw the killing fields and fresh corpses exuding so much blood that they floated down a river of it, which flowed into Tiger Wing. They demanded blood. At once, Thida was possessed again and many workers fainted. In another dream, Thida visualised many workers falling in a mass faint. The next morning, she arrived at work and people came to tell her that the night before, they had gone to the toilet and were confronted with blood flowing along the floor. Her co-workers thought that she had a special connection with the ghosts of those who had died in the locality and they immediately fainted. Women such as Thida were mistrusted by management and could lose their jobs.

### Contemporary Disputes

Sometimes, a mass fainting was connected to industrial discord and labour disputes. Sabrina Factory was built by a Taiwanese Christian and within a couple of years, 500 workers fainted in two episodes. Chantha, a junior union official at Sabrina and his senior, Naren, a Chinese-Khmer, told the story.

A popular legend tells that years ago, two young women were raped and murdered and their corpses discarded under a Bodhi tree at the site. During the Khmer Rouge period, victims were executed under that tree, and after liberation in 1979, pimps from the local brothel would dispose of non-paying clients there. The place became littered with ghosts, their groans audible at night. Black dogs rushed into the tree and vanished. In 2000, the Taiwanese owner felled the tree while clearing the site for his factory, leading to the first mass fainting incident.

Naren believed that because he had failed to pay his respects to ‘White Young Woman’ and ‘Black Young Woman’, they had triggered an episode of mass fainting. He recounted the story of KS, a part-time medium who also worked at Sabrina Factory. She had recurrent dreams of good spirits congregating at the factory to protect it, and one night, she dreamt that the good spirits had been ‘tied up’. She warned Naren that this meant the factory would go bankrupt, so he invited monks to sprinkle lustral water there, which freed the good spirits to return by boat to guard the factory.

Remarkably, Chantha and Naren each had an identical dream in which they complied with ‘Old Lady White’s’ request for a spirit house, and they convened the workers to reassure them that she would protect them. By sheer bad luck, however, a pregnant worker had just been killed by a machine on the factory floor, so the workers interpreted the dream, instead, as a portent that her wandering ghost (*kmaoc taay haoŋ*), hungry for blood, would attack them. Chantha and Naren’s efforts backfired and sure enough, a mass fainting occurred.

This account shows how beliefs can trace a line from the origins of violent deaths long before the factory had been established to present-day mass faintings. The final wave in the tide of misfortune and inauspicious death at the factory site was the pregnant worker, killed not only *at* but *by* the factory.

Three types of inauspicious deaths were viewed as triggering episodes of mass fainting. Factories were far too often the sites of fatal industrial accidents. Early one morning at Heart Enterprise Factory, for example, while the workers were entering the gates, a cement-mix truck reversed and, to the horror of the throng, killed a woman arriving for work. A short time later, a co-worker opened the door to the toilet and saw a wandering ghost, which everyone realised was the ghost of the worker that had been killed, and within minutes, 50 workers had collapsed.

Another trigger was a co-worker attempting suicide. The onlookers panicked, fearing that they would be next, which led to a mass fainting. LS worked at Vann Ko Factory. She grew up in an atmosphere of suicide, the *mṛityu* spirits having induced her family to try to hang themselves. LS’s father-in-law said the doctor warned him that before starting medical treatment, she must get clearance from her traditional healer in case the spirits were still there. The healer noted that she was ravenous, a sign that she was possessed by a hungry spirit, and she was successfully treated by a ritual that involved the pouring of lustral water.

One contemporary tragedy can lead to another. In November 2010, in what Prime Minister Hun Sen depicted as the greatest tragedy that Cambodia has experienced since the Khmer Rouge, 347 people died in a human stampede on the Diamond Island Bridge (Hsu and Burkle 2012).

Heart Enterprise Factory was built on land owned by the spirit known as ‘Honourable Mister Twin’ (*neak taa cah srok plʊəh*) and on which, there was a *ficus religiosa* tree. People paid their respects by building a small hut nearby, but the Chinese owner did not allow it to remain. When the mass fainting occurred, the Chinese managers called LT, a lay Buddhist officiant, to rush monks to the factory and organise an offering to the ‘Landlord of Water, Landlord of Earth’. They offered the head of a pig, some chicken, a bunch of areca, cigarettes and fruit, and the manager offered seven incense sticks.

Everything seemed fine and the workers got the weekend off, but just as they were about to leave, a *kmaoc ʔaʔnaatʰaa* from the stampede disaster on Diamond Island Bridge wandered in and possessed a worker, who fell into a trance at the feet of the monk. The clinic brought out a stretcher, but the officiant stopped them and recited a magical stanza and sprinkled water, and she spoke—on behalf of the ghost in her—‘My name is Diamond Lady. I come from Diamond Island!’ The doctor remonstrated that, no, she had fainted because of toxic fumes in the factory and warned the officiant to stop interfering because unless she immediately got to the hospital, she could die. The officiant told the doctor he was wrong and that the worker’s pulse showed the features of someone possessed, in this case, by a ghost from Diamond Island. By now, a band of more than 100 ghosts had arrived and possessed two other workers, triggering scores of workers to faint over the next 3 days. One of the possessed workers cried, ‘We came from Diamond Island because we are starving and parched!’ But no further food was forthcoming, so the ghosts abandoned the woman, who recovered. The doctor ran after her to check her pulse and finding it then to be normal, believed the healer.

This scenario illustrates how people perceived the mass panic at the factory to be triggered by an earlier national tragedy in which many factory workers on their day off were trampled to death in a stampede, and in effect, they recreated the national disaster on a local scale. The ceremonies performed after the stampede did not help the *nameless* workers who had been killed. Mail posted without a named addressee ends up at the dead letter office. In similar fashion, offerings to the unnamed were lost and the ghosts of the unidentified dead became wandering spirits. The night after the Diamond Island ghost left, LT had another dream in which ‘Mister Twin’ wailed, ‘I am alone! There were many invaders from Diamond Island. I could not defend the land against them all!’ The dream told LT that ‘Mister Twin’ had failed. The villagers were accustomed to seeking protection from him when someone lost a cow, for example, or when a child had fallen ill, but the scale of the Diamond Island stampede was beyond the scope of such routine issues.

The Chinese-speaking Khmer factory manager also believed that ghosts from Diamond Island had caused the mass fainting at his factory, and he knew about the claims of the Cambodian Confederation of Unions for the back pay of the dead workers.

#### Ethnicity and Religion

Ethnicity and religion also made a difference. One Monday morning at Nanguo Factory in Preah Sihanouk province, almost 200 workers fainted. One worker, MST, had a Cham father (KM) and a Khmer mother. She screamed, ‘Oh, the ghosts have come already to take my life!’ and remained in a stupor for 2 days. Her father had her moved to a hospital in Phnom Penh where she continued to scream that she could see the ghosts. Meanwhile, her mother went to an astrologer who diagnosed a ‘violation against the maternal ancestral spirits’, which MST’s father, a Muslim, did not accept.

KM realised that his Khmer wife and her parents had strong beliefs in ghosts, which, as a Muslim, he eschewed in favour of the ‘chemical’ pesticide explanation offered by Union President Chea Mony. He was irate that management blamed the workers. They said that mass faintings ‘always’ occurred on Mondays because workers were hung-over from their weekend revelry. His daughter, however, as a Muslim, would never drink alcohol and because she followed Cham comportment, never went anywhere on Sundays.

As virtually all of the factories were foreign-owned, dissatisfaction was expressed along ethnic lines, often towards the Chinese, who were, after all, the predominant owners. Some owners authorised ceremonies consisting of Chinese rather than local Khmer elements, and they failed to work. The people believed that a mass fainting reflected a clash between Chinese and local Khmer traditions, and perhaps, between the Khmer ‘working class’ and the foreign ‘ruling class’.

#### Industrial Conflicts

The factories simmered with industrial conflicts. A union dispute could spill over into the uttering of an oath, a sacred matter in Cambodia, violations of which might lead to catastrophic consequences. For example, Naren, mentioned above, was a long-standing elected senior member of the Union of Free Workers. His youthful rival, Chantha, coveted Naren’s position and thought him to be a half-breed stooge who helped the Chinese bosses rather than the workers.

An astrologer confirmed Naren’s suspicions that Chantha was the ringleader of the opposition who had set up a rival union. Chantha said that the workers had removed Naren and elected him in his place in early 2012, and Naren’s group had tried to stage a counter-coup. Chantha lost his job. He stirred up his faction and called for a strike.

Then came the fateful declaration of an oath by Naren and his followers who swore that if Chantha was ever reinstated, they would cut off their arms for dogs to feed on. To their chagrin, Chantha appealed and got his job back, an outcome Naren’s followers thought was prompted by ‘Old Lady White’ and ‘Old Lady Black’ charming the Board.

Despite the reinstatement, they did not cut off their arms and within days, ‘Old Lady White’ and ‘Old Lady Black’ retaliated and there was an episode of mass fainting. The spirits had entered a worker and, by controlling her speech, demanded that the owner offer to build a spirit house as compensation. A devout Christian, the owner did not believe in spirits, but the union persisted and he let them build the spirit house. Business prospered and the owner, now convinced, paid for the continued upkeep of the hut. Episodes of mass fainting declined.

The violation of an oath, a ‘wrong vow’ (*khoh sɑmbɑt*), was so serious that one vow-taker declared, ‘If I break this vow, may five generations of my children feel the effects’. Breaking a vow can provoke massive retaliation and the violators or their descendants can become ill as a result of being ‘defeated by vow’ (*caɲ sɑmbɑt*). Mass fainting was viewed as an attempt by the spirits to regulate the conflict.

## Discussion

Globalisation and the advent of the neoliberal economy in countries such as Cambodia bring in their wake what Root (2009) has called ‘hazarding health’ in a ‘risk society’, with an army of women facing multiple risks as they become garment workers. This article suggests that mass fainting is an expression of this new dynamic in a once traditional society, and the results provide an insider’s view of its cultural tapestry. With few exceptions, such as a report by Piñeros et al. (1998) of an epidemic of ‘collective conversion and dissociation disorder’ in response to cultural change in the indigenous Embera of Peru, the cultural meaning of mass reactions to conflicts arising from modernisation has seldom been identified.

There is a rich texture to the explanations for mass fainting that go far beyond the ‘occupational health’ concerns normally raised. The factory site is pivotal. As with other cultural groups in Southeast Asia, any infraction of conduct, past or present, or disruption to the relationship with the spiritual landlords, can lead to consequences for one person or many.

Firth (1967), in his observations of ritual and drama in Malay spirit mediumship, elaborates on what happens to the *spectators* and this is central to understanding the Cambodian workers who began as spectators and ended up playing a dramatic, central role. Mass fainting and states of possession are mediated by the tutelary masters of the factory and by the spirits of those who have had their lives cut short by accidents, execution, or suicide.

The results of this study show the implications of the non-disclosure of a landholding’s legacy of ghosts. This is salient even in modern Cambodia where a prospective buyer may call in an astrologer or *kruu* to see if there is a risk of misfortune. The path to an area associated with ghosts is known as the ‘Spirit Road’ and people are loath to build there or to occupy the land.

### Spirits

Mary Keller noted that in Malaysia in 1979, a woman became possessed in a Japanese-owned factory and ‘her supervisor recounted that the workplace used to be a burial ground, implying that the shop floor was likely to be haunted by angry spirits and that women who had weak constitutions needed to be spiritually vigilant so they would not be possessed’ (Keller 2002). The same is true in Cambodia.

The results of this study illustrate the power of the local guardian spirits. There is abundant literature on guardian spirits in Cambodia (Davis 2013; Guillou 2012; Ledgerwood 2012). The term ‘*neak taa*’ is a benevolent celestial being that normally takes care of people (from Sanskrit, *rakshā* means ‘protection’) and resides in the same local territory as the people (Commission des Moeurs et Coutumes Cambodgiennes 1974; Sal 1966, pp. 24–25). Davis (2013) depicts the guardian spirits as gaining history through possession, so that they become known individually and change over time. Our results show how the names of particular guardian spirits carry unconscious powerful messages. A sugar palm struck by lightning and left ‘decapitated’, for example, is a sign of evil and misfortune. Subsequently, there is the notion that the boundary where forest and cultivated fields meet is a point of demarcation and danger where people may be attacked.

I have referred in an earlier paper to the post-canonical Sutta Nipāta commentary (Jayasekera 1985) as providing a Buddhist prototype for illness following disregard for the spiritual owners of the land (Eisenbruch 2017). Five hundred monks went into a remote forest where they chose a big tree under which to meditate. Little did they know that they had chosen the location of the malevolent Asurakaya spirits. The next morning, the monks went looking for food. The spirits climbed their tree, expecting that the monks would depart but after a week, they were still there. Angered, they transformed themselves into ‘haunting ghosts’ with big eyes, making piercingly high-pitched sounds and howling like dogs. The terrified monks ran to the Buddha, who ordered them to return to the tree and recite ‘Karaṇīyamettā Sutta’ to teach the spirits about loving-kindness. At once, they stopped haunting the monks. Like the spirits displaced by the factories, the spirits of the trees had been displaced of their home and stood aside waiting for the 500 monks to move on. The monks disregarded the spiritual ownership of the land and became ill—their brains ‘smothered’ by the ‘visions, noise and stench’ (Buddharakkhita 1989).

The issue of mass fainting also reflects social and class struggles over land. Economic developments such as the garment industry were a part of the globalisation that went hand in hand with the loss of individual landholdings and the capacity of people to eke out a living for their families. The parallel is that the garment industry was also seen as having robbed the ‘Landlord of Water, Landlord of Earth’ of their spiritual territory. The little people had no voice with which to directly make their protest, but they could make themselves heard, one might say, through the social protest on the part of spirits that ‘caused’ mass fainting. The conflict is solved by agreeing to their conditions, for example, when the foreign owner of Tiger Wing Factory built an altar for the ‘Landlord of Water, Landlord of Earth’.

Traditionally, in conflicts among children in a neighbourhood, the respective parents would punish their own children rather than seek to establish who started the fight. Similarly, ancestral spirits should not attack their innocent descendants for the wrongs committed by their parents, but do so as a dire warning to get the parents to change their ways. The innocent bystanders, those who were sceptical or non-believers, who scoffed at the tradition, were the ones who were afflicted by the actions of the supernatural ghosts and spirits, rather than the wrongdoers. As the traditional saying goes, ‘Ghosts and spirits attack the ones who laugh rather than the wrong-doers’. Spirits, however, do attack the weak, as expressed in the saying, ‘When your Zodiac House is low, a bad spirit will tread on you’ (*yii ʔun* is a Chinese loan word meaning ‘opportunity’ or ‘fortune’ that is combined with *dac* for ‘lowered’ and *?aareak coan*, meaning the ‘*?aareak* spirit that trod on’). This is an injustice that, in the example from Eternity Global Sporting Factory, was highlighted when the supreme deities brought together all the humans of the middle world and the local spirits of the lower world to resolve their conflict, to stop blaming one another, and to come together for the common good.

### Ethnicity

The factory owners are Chinese, Taiwanese, Korean and Malaysian, and inter-ethnic conflicts between the bosses and the local workers arise. Chinese factory managers reportedly abscond, leaving Cambodian workers without pay, while Chinese managers who stay reportedly live in fear of angry factory workers (Chong 2017). The findings of this study show repeated examples of the tensions between the workers and the owners.

We have reported on the tone of the foreign ‘languages’ spoken by the spirits. It did not matter whether the language was *really* Chinese, that was the brand, an allusion to the ‘Chinese-ness’ of so many of the factory owners. (Actually, many were Chinese, Taiwanese, Korean and Malaysian, and some were even Khmer.)

There is another dimension to the Chinese-Khmer story. Davis (2013) has highlighted the extravagant display of ethnic Chinese identity that, he observed, dominated guardian spirit possession rituals in contemporary Cambodia. The ‘Chinese’ ethnicity of several factory owners seems relevant. A guardian spirit can possess innocent people, such as factory workers, not just those who explicitly violated their rules, traditions, and rituals (Eisenbruch 2017). Chinese factory owners, likewise, are in a special position because they have an affinity for the notion of guardian spirits, which ordinary people saw as instrumental in causing mass faintings.

The example of the annual ceremonies at the M &V Factory, which failed to prevent mass faintings, show how Chinese rituals might satisfy the ancestral spirits of the Chinese owners but were useless in preventing the mass faintings of Khmer factory workers. Worse, mass faintings occurred year after year in the wake of the Chinese ceremonies. The Chinese offering is performed, in part, to ask the ‘Head of the Water’, literally, the river upstream, to turn off the source of flooding. It also propitiates the Chinese ancestral spirits, or *maa kong*, that emerge at that time, a month before the Khmer *pcum ben* or ‘All Souls Ceremony’.

The prominence of ‘Chinese’ and other ‘foreign’ languages uttered by the possessed workers signal the inter-ethnic and class struggles between the workers and the bosses. Ethnic war traditionally was levelled against the Thai and the Vietnamese, evidenced by the saying, ‘When you descend into crocodile-infested water, you ascend onto land where the tiger awaits you’. Now, however, multinational sources provide wages for desperately poor workers, but at a cost, and the resulting tension is expressed as tension between the local spirits and the foreign bosses.

### Inauspicious Deaths

The Khmer Rouge did not bother to seek permission from the spiritual proprietors before executing people. The workers feared some factory sites to be inauspicious places where the ghosts of those who had died prematurely in terror and at the hands of violence would lurk to avenge their deaths on anyone who crossed them. A generation later, the construction of the factories completed the provocation (Eisenbruch 2017). All these associations were linked, metaphorically, as in the example of Thida’s bad dreams and omens involving rivers of blood literally flowing in the toilets of the terrified workers. This and other dream narratives reported in this article show the importance of communication with the dead (Stewart and Strathern 2003) in making sense of mass faintings.

The role of the killing fields leads us to a broader question regarding inauspicious death. Given the stain of inauspicious deaths in Cambodia, it is as if grief is daisy-chained from one tragedy to the next. A recent trauma, such as the Diamond Island disaster, for example, can lance much older wounds of grief stemming from the Khmer Rouge era and can feed newer traumas such as the mass faintings. Field et al. (2014) revealed the place of Prolonged Grief Disorder (PGD) among mothers using an example of a daughter who was killed during the Diamond Island stampede. Such a loss is more difficult to integrate than expected loss, and the present study shows how, through the widespread belief in haunting ghosts, this effect radiates well beyond the immediate family, affecting several hundred unrelated garment workers and leading to mass fainting.

In my earlier article (Eisenbruch 2017), I argued that inauspicious and premature death is thought to trigger mass fainting, and it is regarded as particularly dangerous in Theravada Buddhist countries, including Cambodia (Guillou 2012; Holt 2012). It is said that the dead are ‘gone, but not departed’ and ‘are active members of the community’ (Williams and Ladwig 2012). The haunting ghosts (‘visitors from Hell’ as Ladwig (2012) put it in the case of the Lao Buddhist festival) render the land dangerous to walk on. There is a rich texture to such ghosts, known as *Asurakaya*, meaning ‘invisible body’ (Filliozat 1951). The Asura lost their chance to enjoy their belongings and, therefore, remain near the location of their corpses, haunting people who intrude on their territory and disturb them when they are busily devouring corpses, and the Asura demand compensation offerings in exchange for ‘protection’. Wandering or homeless ghosts (Pali *āvāsa* for habitat or domicile) are related to the location and may enter a victim’s body in search of lodging. This is in contrast to a person who dies with no surviving relations and becomes a ‘vagrant ghost’ or *kmaoc ʔaʔnaatʰaa* that cannot rest because there is no one to take care of his or her spirit needs—they become like ‘street children’ of the other world and need a home. If a person dies of accidental causes, such as drowning, the ghosts are ‘ghosts that pull your feet’ (*kmaoc cəəŋ kɑɑp*) or lie in wait in whirlpools to seize reborn swimmers, leading to a string of ‘accidental’ deaths. Finally, the ghosts of the dead can move from one tragic death to the next, as in the ghosts of the Diamond Island mass stampede that crossed over to the factory, the site of a mass fainting.

The belief in haunting ghosts, or *kmaoc taay haoŋ*, is widespread throughout Cambodia, Thailand and Laos (Hinton et al. 2005; Johnson 2013; Patamajorn 2008). They detest having to let go of their worldly possessions for someone else to enjoy and are called ‘ghosts who protect the territory to which they are so attached’ (*huǝŋ* = fighting to take possession and seize; *haeŋ* = all dried up, extremely vulgar). People are terrified of burial pits, for example, as illustrated in popular media reports about Kampong Chhnang’s famed ghost house (Carden and Shelton 2008), or by the horror movie *The Haunted House* (Anonymous 2006).

We have reported cases in which people believe that these ghosts caused mass faintings. Cases in the vicinity of Sabrina Factory show the prominent role of high-ranking ‘haunting ghosts’ that evolved into figures of veneration. ‘Madame Grandma Black’ originated in the sea near Koh Kong and moved inland to Pich Nil Mountain. Her popularity grew as far afield as the vicinity of Sabrina Factory, where statues and shrines were erected near homes and market places, allowing the locals to make offerings and thereby gain her protection.

Work in the garment industry can be fatal, and we have reported cases in which the *mṛityu* ghosts induced workers to commit suicide in the factory. The Indo-European root word ‘mort’ for death comes from the Sanskrit word ‘*mṛityu*’, which is the same word in Khmer for the ghost of a person who has committed suicide. In a ‘*mṛityu* illness’, the ghost possesses the victim and withdraws some of their 19 souls. The victim feels restless and broody (*muə mav*) and may wander into the dark forest to hang themselves (Miech 2001, pp. 181–185). In the factory setting, the spirit of the deceased avenges the intruder’s infraction by commanding the worker to kill herself and, in turn, her ghost will seek to make others follow suit.

## Conclusion

There are many questions in understanding current events in Cambodia, and with the coming closure of the international tribunal (ECCC) there is renewed interest in coming to terms with the mixture of beliefs that govern Cambodian actions concerning rights, responsibilities and social action. This article helps to address this interest. The fainting in the garment industry is a phenomenon that is not understood—and the fact that it receives modest attention should be seen as a sign that many social interactions in Cambodia are still so poorly understood.

This study suggests questions for which there are as yet no answers. Is mass fainting a ‘new’ and unique phenomenon in Cambodia? Does it simply echo what has been seen decades earlier in other newly industrialised settings such as Malaysia? Or is it a reinvention of similar and culturally embedded group idioms or mass possession phenomena in Cambodia before this contemporary ‘epidemic’.

This study captures the flavour of the popular attributions of mass fainting. Some involved violations against the local tutelary or guardian spirits who were displeased and offended that their permission had not been sought before factories were built or because they were constructed on sites thought to be associated with inauspicious deaths (the Khmer Rouge killing fields or the sites of suicides or fatal industrial accidents). Additionally, bad conduct by the employees or their management would also provoke retaliation by the local spirits. A conflict within the supernatural realm is a metaphor for a conflict that affects the lives of workers, with mass faintings sometimes perceived as spiritual demands for industrial and social justice.

The expression of conflict, whether in the human or supernatural realm, seems central. Cambodians have traditionally said that the ‘*deva* have eyes’, meaning that if people cannot achieve social justice through the rule of law, the deities and spiritual protectors will come to their aid and bear witness on their behalf. This article it clearly points out the role of the supernatural in relation to public health issues, not only in theoretical assumptions, but in the concrete, daily lived reality of many Cambodian people.

Mass fainting could be seen as a culturally coherent form of social protest, which would allow civil society and the garment industry to be better equipped in their response. The usual answer from rights-activists to suggestions like this—taking culture seriously—is that it would undermine the efforts made to come to more healthy working conditions. I show the discrepancy between the discourse of parties that operate in the ‘neoliberal worldview’ (factory owners, government, workers associations, labour unions) on the one hand and many of the Khmer workers in the garment industry on the other. Once mass fainting is seen as a culturally coherent form of social protest, civil society and the garment industry will be better equipped to respond in ways that promote the human rights of the workers, minimise the need for further episodes of mass fainting and improve economic productivity and human happiness. We are left with the question: do the workers in countries such as Cambodia need to be drawn into the ‘modern world’ in order to deserve social justice? Or could social justice include traditional beliefs?

The public health messages about the causes of mass fainting no doubt are of value in reducing risk and no doubt people respond to health education on lack of nutrition or exposure to toxins, but public health messages should be articulated with cultural insights such as those provided by the present study. In responding to communities affected by mass events, current public health approaches need to enlarge an encompass spiritual aspects of care, but this must be done in a culturally responsive manner. Policy makers and providers should develop the capacity to identify and work with the ways in which an affected community, in its response to minimise suffering and mishap, draws on its local cosmologies including magical or supernatural agency. The bigger the scale of the disaster, the greater the role of cultural eschatologies and millennial tendencies in response to the threat. Public health interventions should fit with and build upon cultural understandings of the mass events that drive people’s behaviour. Interventions by monks and healers help restore hope and confidence. Buddhist and healing therapies could ameliorate continuing personal and work tensions and reduce the likelihood of further episodes of mass fainting.
